# Genetic Diversity, Phylogenetic Lineages, and Antigenic Features of the PRRSV-1 GP5 Gene in China

**DOI:** 10.3390/microorganisms14091963

**Published:** 2026-09-05

**Authors:** Fang Liang, Tianyuan Nie, Peixiu Lin, Huawei Li, Ruining Wang, Xuyong Zhao, Lin Wang, Keshan Zhang, Yaqiong Ye, Mengmeng Zhao

**Affiliations:** 1 School of Animal Science and Technology, Foshan University, Foshan 528225, China; 15726370117@163.com (F.L.); arceev587@gmail.com (T.N.); 19865918906@163.com (P.L.); zks009@126.com (K.Z.); 2 College of Veterinary Medicine, Henan University of Animal Husbandry and Economy, Zhengzhou 450046, China; centrosome@126.com (H.L.); 80882@hnuahe.edu.cn (R.W.); zhaoxy6868@163.com (X.Z.); 3 Institute of Cancer Sciences, University of Glasgow, Glasgow G12 800, UK; lin.wang@glasgow.ac.uk

**Keywords:** PRRSV-1, *GP5* gene, genetic variation, phylogenetic evolution, antigenic epitopes

## Abstract

Porcine reproductive and respiratory syndrome virus (PRRSV) poses a significant threat to the global swine industry. In this study, the genetic diversity, evolutionary dynamics, and key antigenic characteristics of the PRRSV-1 GP5 gene in China were systematically investigated. A total of 114 Chinese PRRSV-1 GP5 sequences, isolated between 1999 and 2024, were analyzed alongside global reference strains. The nucleotide and amino acid sequence similarities ranged from 60.03% to 100% and from 48.72% to 100%, respectively, with mutation and deletion hotspots concentrated in the N-terminal hypervariable region. Phylogenetic analysis further classified the Chinese strains into four major lineages: Amervac-like, BJEU06-1-like, HKEU16-like, and NMEU09-1-like. Notably, a preliminary phylogenetic observation indicated that several recent Chinese PRRSV-1 isolates clustered topologically close to PRRSV-2 strains. However, as this finding is based solely on a single-gene tree and has not been validated by formal tests for convergent evolution, homoplasy, or selection pressure, it should be interpreted with caution and warrants further investigation using whole-genome data. A Bayesian evolutionary analysis estimated the evolutionary rate at 1.94 × 10^−3^ substitutions per site per year and the time to the most recent common ancestor at approximately 1910. No statistically significant recombination events were identified within the GP5 gene. The transmembrane topology of GP5 was highly conserved, whereas B-cell linear epitopes were enriched in the N-terminal extracellular region. This study establishes a useful molecular foundation for understanding PRRSV-1 evolution in China and offers valuable insights for the development of broad-spectrum vaccines and effective control strategies.

## 1. Introduction

Porcine reproductive and respiratory syndrome virus (PRRSV), the etiological agent of porcine reproductive and respiratory syndrome (PRRS), predominantly infects swine and is responsible for major reproductive and respiratory disorders. Infection in pregnant sows is commonly associated with reproductive failure, including abortion, stillbirth, and fetal mummification, whereas pigs of all ages may develop respiratory symptoms [1,2,3]. PRRSV is classified into two distinct genotypes: PRRSV-1 (species Betaarterivirus europensis [formerly Betaarterivirus suid 1], European type, prototype Lelystad virus) and PRRSV-2 (species Betaarterivirus suid 2, North American type, prototype VR-2332), which share approximately 50–60% nucleotide sequence identity [4,5]. Following the first isolation of PRRSV in Europe and North America in the early 1990s, PRRS subsequently became endemic in many countries worldwide [6,7]. In 2006, outbreaks of highly pathogenic PRRS (HP-PRRS), characterized by high fever and elevated mortality across age groups, were reported in China and rapidly disseminated to several Asian countries [8]. Morbidity and mortality during these outbreaks were estimated at 50–100% and 20–100%, respectively. Between 2006 and 2007, approximately 40 million pigs were depopulated in China, resulting in severe economic losses to the swine industry [9]. Despite extensive control efforts, PRRS has remained difficult to control globally, largely due to factors such as pronounced genetic diversity, virus-induced immunosuppression, and the continuous risk of reinfection in negative herds [10,11]. As a result, PRRS continues to pose a sustained and substantial threat to pig production worldwide.

Although the epidemiological features and genetic evolution of PRRSV-2 in China have been intensively investigated, molecular epidemiological information on PRRSV-1 remains incompletely understood. In recent years, with the expansion of international trade and increased cross-regional movement of live swine, PRRSV-1 has been detected in an increasing number of provinces in China. Its detection rate has increased consistently, and regional clustering has been observed particularly in parts of Southern, Eastern, and Northeastern China [12,13]. These findings indicate that PRRSV-1 may no longer represent solely an “imported” pathogen; instead, local transmission chains are likely to have been established in certain regions. Moreover, co-infection and co-circulation with PRRSV-2 may be occurring, thereby elevating the risks of viral recombination, antigenic drift, and immune interference.

Glycoprotein 5 (GP5), a major structural protein of PRRSV, participates in virion assembly and host–cell recognition. Importantly, principal neutralizing epitopes are carried by GP5, rendering it a key target for vaccine development and immunological surveillance [14,15]. Nevertheless, systematic and longitudinal investigations of the PRRSV-1 *GP5* gene in China remain scarce. Previous studies have largely focused on PRRSV-2 or have been limited to localized outbreaks and short-term epidemiological observations of PRRSV-1. Consequently, critical scientific problems—such as the long-term temporal evolutionary patterns and geographic distribution of PRRSV-1, its specific evolutionary rates within Chinese swine populations, and the spatial structural consequences of accumulated GP5 mutations—remain largely unresolved. Given the emerging epidemiological significance of PRRSV-1 and its extensive co-circulation with PRRSV-2, it is important to systematically investigate the genetic and antigenic variability of the GP5 gene that may underline potential immune escape. However, we emphasize that direct assessment of immune escape from currently available vaccines requires functional and immunological experiments, which are beyond the scope of this sequence-based study.

The PRRSV genome is approximately 15 kb in length and contains at least 11 open reading frames (ORFs) [16]. Among them, ORF5 encodes the major envelope glycoprotein GP5, which forms a heterodimer with the M protein and plays a critical role in viral attachment and entry into host cells via receptor-mediated endocytosis [17]. Importantly, ORF5a, a small overlapping gene located within the ORF5 region, encodes a conserved arginine/glutamine-rich peptide that imposes structural constraints on the GP5-encoding frame, partially explaining the paradox of high sequence variability in the N-terminus alongside maintained structural stability [18,19]. Given that GP5 carries the principal neutralizing epitopes, its genetic and antigenic evolution directly impacts the efficacy of current vaccines and the emergence of immune escape variants [14,20].

To address these knowledge gaps, a comprehensive analysis of the PRRSV-1 GP5 gene in China, encompassing a 25-year timeline (1999–2024), was performed in the present study. Although conventional serological and neutralization assays are indispensable for functional validation, they are low-throughput and cannot be applied retrospectively to historical strains. In contrast, large-scale in silico analyses integrating phylogenetic inference, Bayesian coalescent models, and advanced structural prediction (AlphaFold3) enable the systematic retrospective characterization of long-term evolutionary trajectories and antigenic trends at the population level. This approach is therefore uniquely suited to bridge the gap between sequence surveillance and functional hypothesis generation, providing a critical foundation before targeted experimental validations are undertaken. Specifically, the genetic diversity and phylogenetic lineages of Chinese PRRSV-1 isolates over the past two decades were delineated. Furthermore, the evolutionary rate and temporal origins of the GP5 gene were estimated using robust temporal models, and the distributions of major mutation sites, glycosylation patterns, and antigenic epitopes within the GP5 spatial structure were systematically characterized. Through this multifaceted approach, the molecular and structural bases of PRRSV-1 evolution in China were elucidated, providing an essential theoretical and technical foundation for broad-spectrum vaccine optimization and the development of targeted, precision-based control interventions. However, all analyses in this study are based exclusively on the GP5 gene. While GP5 is a key target for immune surveillance and vaccine development, a single-gene approach cannot capture whole-genome evolutionary dynamics, nor can it reliably infer recombination or population-level transmission histories. Therefore, our conclusions are limited to the GP5 locus and should be interpreted within this context.

## 2. Materials and Methods

### 2.1. Strain Collection

**Figure 1:**
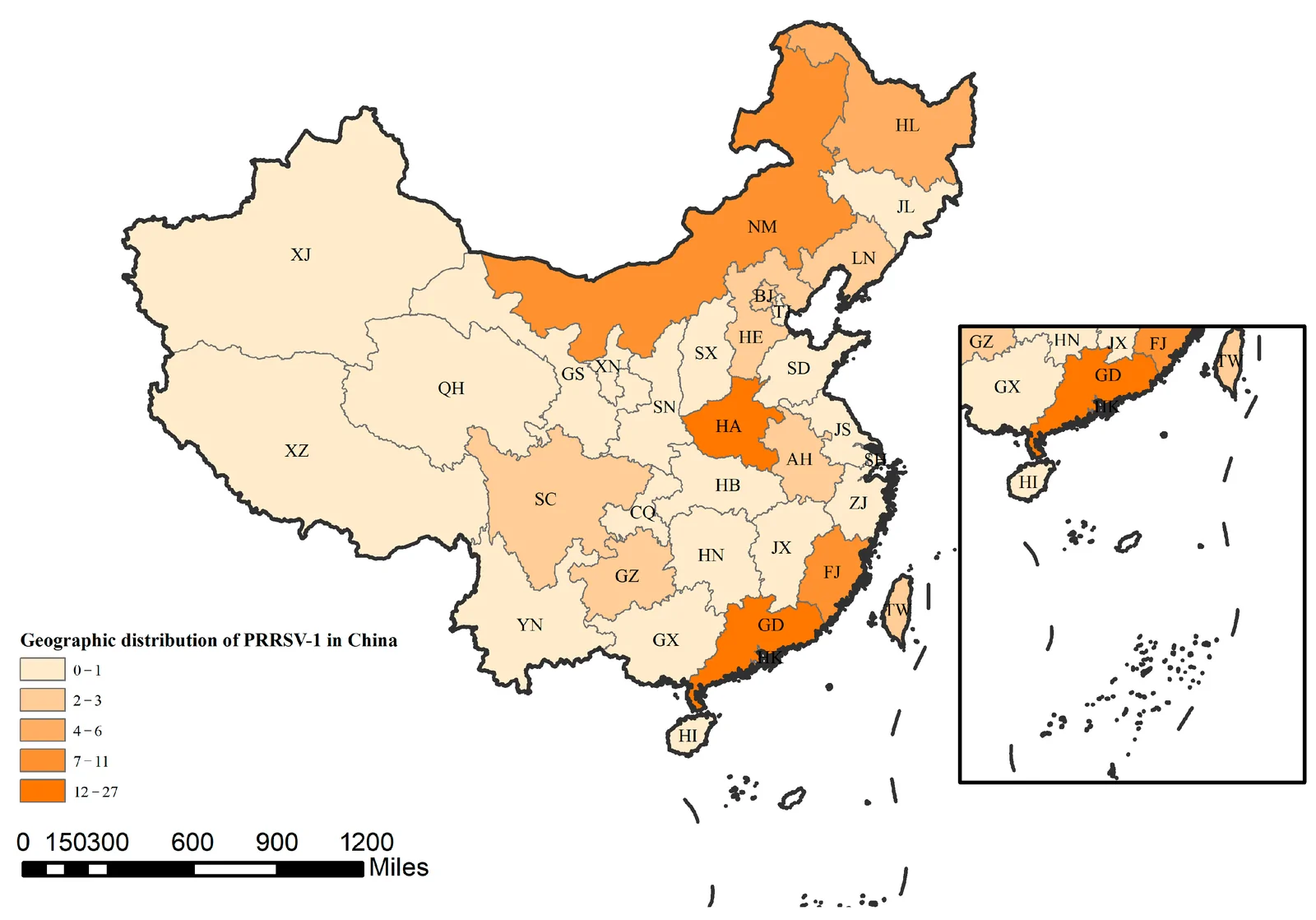
Geographic distribution of PRRSV-1 in China. In this province, different shades of color are used to represent the relative abundance of strains: darker shades indicate a greater number of strains, while lighter shades indicate fewer strains. The detailed province-level information and GenBank accession numbers for each strain are available in Table S1.

In this study, a total of 114 PRRSV-1 GP5 sequences isolated from China were collected from the GenBank database (Table 1). To comprehensively analyze the genetic variations in the PRRSV-1 GP5 gene over time and among different strains, and to facilitate the differentiation between domestic and foreign PRRSV-1 strains, 19 overseas PRRSV-1 GP5 sequences and 16 PRRSV-2 GP5 sequences were also included (Table 1). The geographic distribution of all Chinese-origin GP5 sequences described above is illustrated in Figure 1. The geographic distribution of all Chinese-origin GP5 sequences is illustrated in Figure 1, and the detailed province-level information for each strain is provided in Table S1.

### 2.2. Sequence Analysis of the PRRSV-1 GP5

In this study, nucleotide and amino acid sequence similarities of 114 PRRSV-1 GP5 sequences were analyzed. All sequences were trimmed to a common length of 204 aa for subsequent analyses. The coding sequences (CDs) of the 114 PRRSV-1 GP5 sequences were retrieved from the NCBI website (https://www.ncbi.nlm.nih.gov/ (accessed on 10 September 2025)). These sequences were processed into nucleotide sequences using the EditSeq module of DNAStar software (DNASTAR, Version 18.0, Madison, WI, USA) and were subsequently translated into amino acid sequences. All sequences were aligned by the Clustal W method within the MegAlign module, and the nucleotide sequence alignment results were generated. Similarly, for amino acid sequence analysis, the sequences were translated and processed using the Clustal W method to produce the corresponding alignment results.

### 2.3. Phylogenetic Analysis

A phylogenetic tree was constructed from 114 PRRSV-1 GP5 sequences (Table 1) using the Neighbor-Joining method with 1000 bootstrap replications in MEGA software (Version 12, Mega Limited, Auckland, New Zealand). Additionally, 19 PRRSV-1 and 16 PRRSV-2 GP5 nucleotide sequences were selected, which included classical strains and vaccine strains from different countries and time periods. The resulting phylogenetic tree was subsequently annotated using the online tool The Interactive Tree of Life (https://itol.embl.de (accessed on 20 September 2025)).

### 2.4. Bayesian Evolutionary Analysis

To investigate the temporal dynamics and evolutionary rate of the PRRSV-1 GP5 gene in China, a Bayesian phylogenetic analysis was performed using the 114 Chinese PRRSV-1 GP5 coding sequences collected from 1999 to 2024. Sequences were aligned using the Clustal W algorithm implemented in MEGA12 (Version 12, Mega Limited, Auckland, New Zealand) and manually trimmed to ensure uniform length and positional homology. The final alignment was converted to NEXUS format and prepared for analysis using BEAUti v1.10.4.

The best-fitting nucleotide substitution model was determined based on the Bayesian Information Criterion (BIC) implemented in jModelTest v2.1.10. The GTR  +  I  +  Γ model was identified as the optimal substitution model for the dataset. A strict molecular clock model was calibrated by incorporating the sampling dates (year of isolation) for each sequence as tip dates. A Bayesian Skyline model, assuming a piecewise-constant demographic history with 10 grouped intervals, was employed to estimate changes in effective population size over time.

Posterior distributions of parameters were estimated using the Markov chain Monte Carlo (MCMC) method implemented in BEAST v1.10.4. Two independent MCMC runs were conducted for 200 million generations, with sampling every 2000 generations. Convergence of all parameters was assessed by calculating the effective sample size (ESS) using Tracer v1.7.2, with ESS values > 200 considered acceptable. After discarding the first 10% of samples as burn-in, the maximum clade credibility (MCC) tree was generated using TreeAnnotator v1.10.4. The evolutionary rate and tMRCA were estimated as the mean values from the posterior distributions of the MCMC runs, with 95% HPD intervals representing their uncertainties. The MCC tree was visualized and annotated using FigTree v1.4.4.

The time-scaled phylogenetic tree was used to infer the evolutionary rate and the time to the most recent common ancestor (tMRCA) for major lineages of Chinese PRRSV-1.

### 2.5. Recombination Analysis

Potential recombination events were identified using seven methods (Chimaera, BootScan, 3Seq, GeneConv, SiScan, MaxChi, and RDP) implemented in the RDP software (Version 4.101). A recombination event identified by any given method was considered statistically significant only if its corresponding *p*-value was less than 0.05. Based on this criterion, a recombination event was considered statistically significant only when it was consistently detected by at least five of the methods. Furthermore, manual verification was performed, as it was noted that the statistical basis for the *p*-values might differ across methods.

### 2.6. Bioinformatic Analysis

#### 2.6.1. Prediction of Protein Domains

Based on the translated GP5 amino acid sequences, predictions of transmembrane domains (TMHMM) were performed for six representative GP5 amino acid sequences using the TMHMM-2.0 online server provided by the Department of Health Technology at the Technical University of Denmark (https://services.healthtech.dtu.dk/services/TMHMM-2.0 (accessed on 15 October 2025)).

#### 2.6.2. Prediction of N-Glycosylation Sites

To determine whether differences exist in the frequency of N-glycosylation sites in the GP5 protein among different PRRSV-1 strains, potential N-glycosylation sites in the amino acid sequences were predicted using the NetNGlyc 1.0 server (http://www.cbs.dtu.dk/services/NetNGlyc/ (accessed on 25 October 2025)) provided by the Department of Health Technology at the Technical University of Denmark (DTU).

#### 2.6.3. Prediction of the GP5 Structure by AlphaFold3

To compare the structural features of GP5 between genotypes, the complete GP5 amino acid sequences of two representative strains—PRRSV-1 strain BJEU06-1 and PRRSV-2 strain VR-2332—were submitted for three-dimensional structure prediction using the official AlphaFold Server web interface (https://alphafoldserver.com/ (accessed on 12 November 2025)), which provides the implementation of AlphaFold3 (v3.0.0; DeepMind, London, UK). Predictions were performed via the online server using default parameter settings (five recycles per model, no template-based modeling, and an MSA depth of 512 sequences). Upon completion of the job, the server returned five predicted models (fold_model_0.cif to fold_model_4.cif). The top-ranked model was selected based on the combined ipTM (interface predicted Template Modeling) and pTM (predicted Template Modeling) scores, following the server’s default ranking criteria (ipTM > 0.8 and pTM > 0.5 as indicators of high-confidence interactions). The selected models were subsequently visualized, and structural figures were generated, using PyMOL (v2.5.0; Schrödinger, Inc., New York, NY, USA).

#### 2.6.4. Prediction of Epitopes

Based on the translated GP5 amino acid sequences, B-cell linear epitope analysis was performed on six representative GP5 sequences using BepiPred-3.0 from the Department of Health Technology at the Technical University of Denmark (https://services.healthtech.dtu.dk/services/BepiPred-3.0/ (accessed on 23 November 2025)). The threshold for predicting B-cell epitope residues was set at 0.1512, with particular focus placed on identifying potential epitopes within the top 20% of high-confidence regions. This bioinformatic analysis was performed to evaluate the presence of linear B-cell epitopes in Chinese PRRSV-1 GP5 sequences.

## 3. Results

### 3.1. Nucleotide and Amino Acid Sequence Similarities of the PRRSV-1 GP5 Gene

To evaluate the genetic divergence of the PRRSV-1 GP5 gene, nucleotide and amino acid sequence similarities were analysed among a total of 133 PRRSV-1 isolates, comprising 114 Chinese strains and 19 non-Chinese strains. The pairwise comparisons revealed that the nucleotide similarity ranged from 60.03% to 100%, with the highest identity (100%) observed between strains FJEU and FJEU13, and the lowest identity (60.03%) found between the Chinese strain GDXNF85-1803 and the Belgian strain SU1-Bel. For amino acid sequences, the similarity ranged from 79.49% to 100%. Complete identity (100%) was detected in several pairs, including FJEU13 vs. FJEU, FJ0602 vs. FJ0603, and NPUST-2789-3W-2 vs. NPUST-2789-3W-5, while the lowest amino acid similarity (79.49%) was recorded between the Chinese strains TZJ2780 and ZZH817. These results demonstrate that, within the PRRSV-1 genotype, substantial sequence heterogeneity exists, particularly in comparisons involving recent Chinese isolates and certain European strains. Detailed pairwise similarity values are summarised in Table 2.

### 3.2. Amino Acid Sequence Alignment

**Figure 2:**
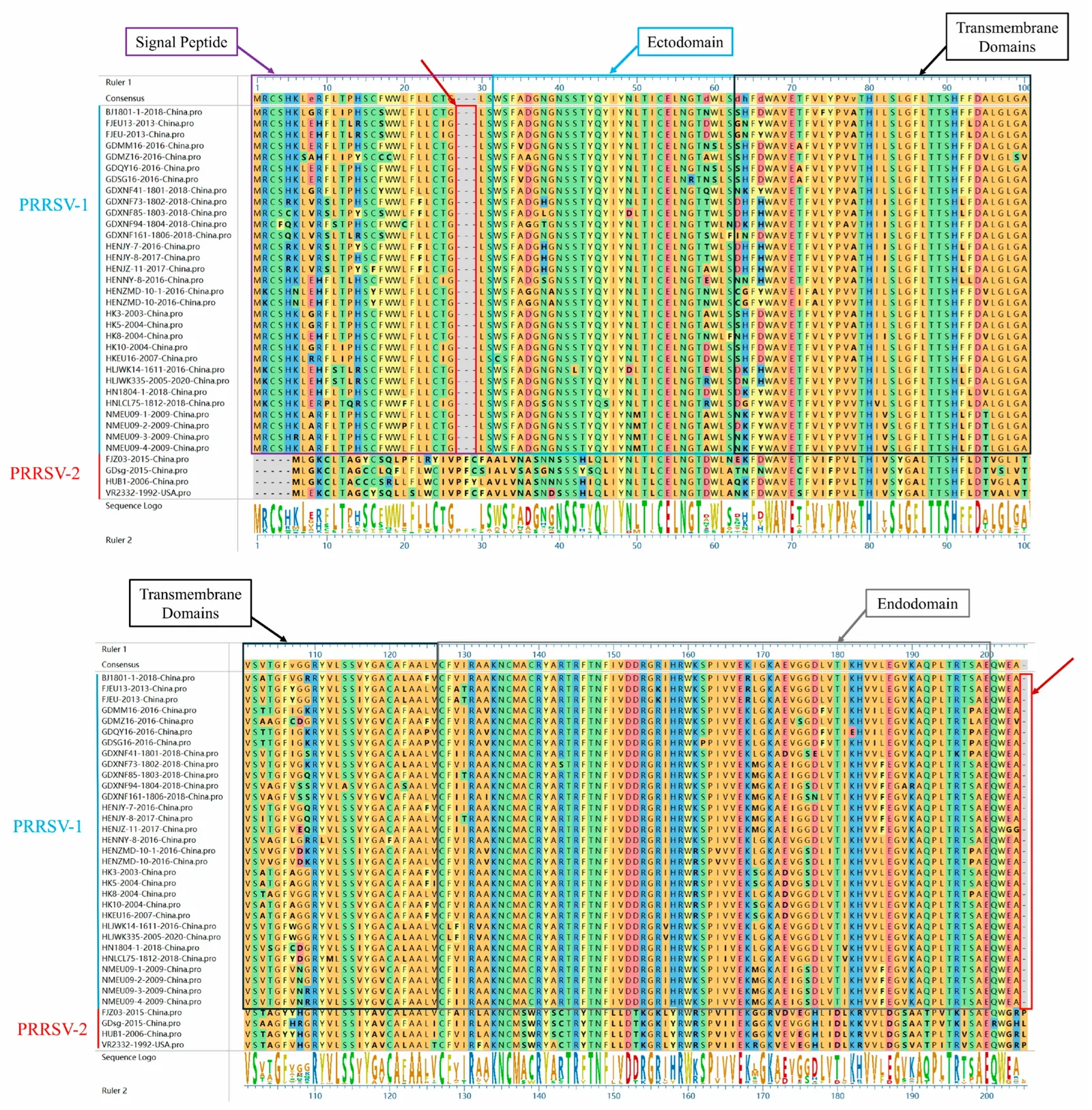
Analysis of the amino acid sequence alignment of GP5. Residues are colored according to Clustal W default schemes. Dashes indicate deletions. The N-terminal hypervariable region is boxed, and key deletion sites (positions 27–29 and 205) are marked with red arrows. The boundaries of major functional domains are indicated based on the PRRSV-1 GP5 topology: signal peptide (SP, aa 1–31), ectodomain (Ecto, aa 32–62), transmembrane domains (TM, aa 63–126), and endodomain (Endo, aa 127–200).

To evaluate the diversity of GP5 amino acid sequences between PRRSV-1 and PRRSV-2, 31 PRRSV-1 and 4 PRRSV-2 GP5 amino acid sequences were randomly selected for a multiple sequence alignment (Figure 2). The results demonstrated that a greater number of amino acid deletion sites were identified in PRRSV-1 GP5 compared to PRRSV-2 GP5. These deletion sites were primarily located at positions 27–29 and 205 of the amino acid sequence. The deletion sites in GP5 were found to be consistent across different PRRSV-1 strains, indicating that stable and conserved deletion events have occurred in the corresponding regions (positions 27–29 and 205) during the evolution of this genotype. This deletion pattern suggests that these sites may be in structurally variable regions or non-critical functional domains of the GP5 protein. While the deletions might not have significantly compromised the essential viral functions, they could potentially influence protein antigenicity or structural flexibility. In contrast, no prominent deletion clusters were observed in the corresponding regions of PRRSV-2 GP5 sequences, reflecting dis-tinct evolutionary trajectories of the GP5 protein between the two genotypes. PRRSV-1 may have utilized the deletion of specific amino acids as an adaptive strategy to either evade host immune pressure or optimize its functional characteristics.

### 3.3. Phylogenetic Analysis Results

**Figure 3:**
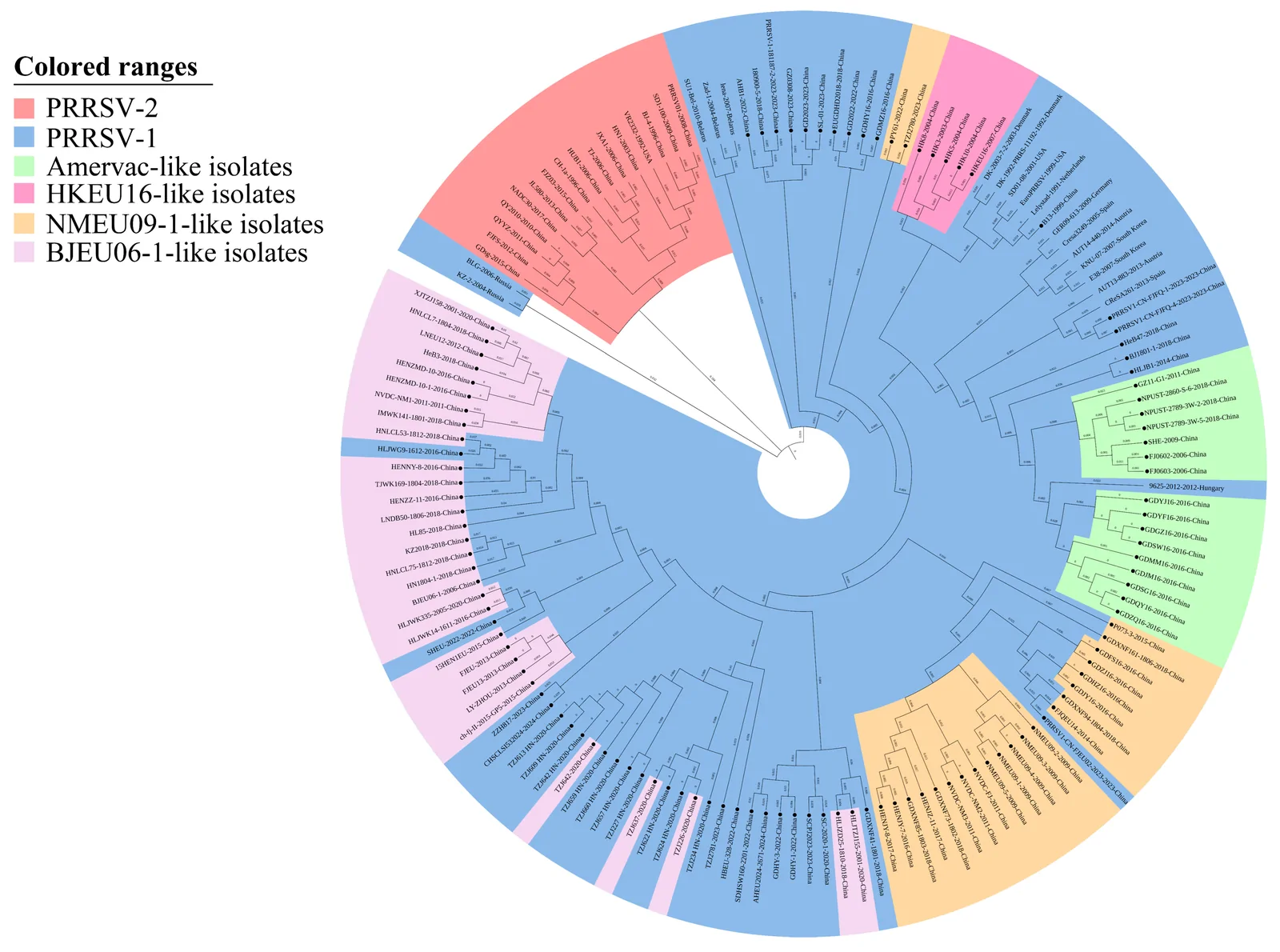
Phylogenetic analysis of PRRSV GP5 gene using the Neighbor-Joining (NJ) method. The tree was constructed based on 133 PRRSV-1 and 16 PRRSV-2 isolates. PRRSV-2 and PRRSV-1 lineages are highlighted with red and blue backgrounds, respectively. Chinese PRRSV-1 isolates are marked with circles (●). Numbers at the nodes represent branch lengths.

**Figure 4:**
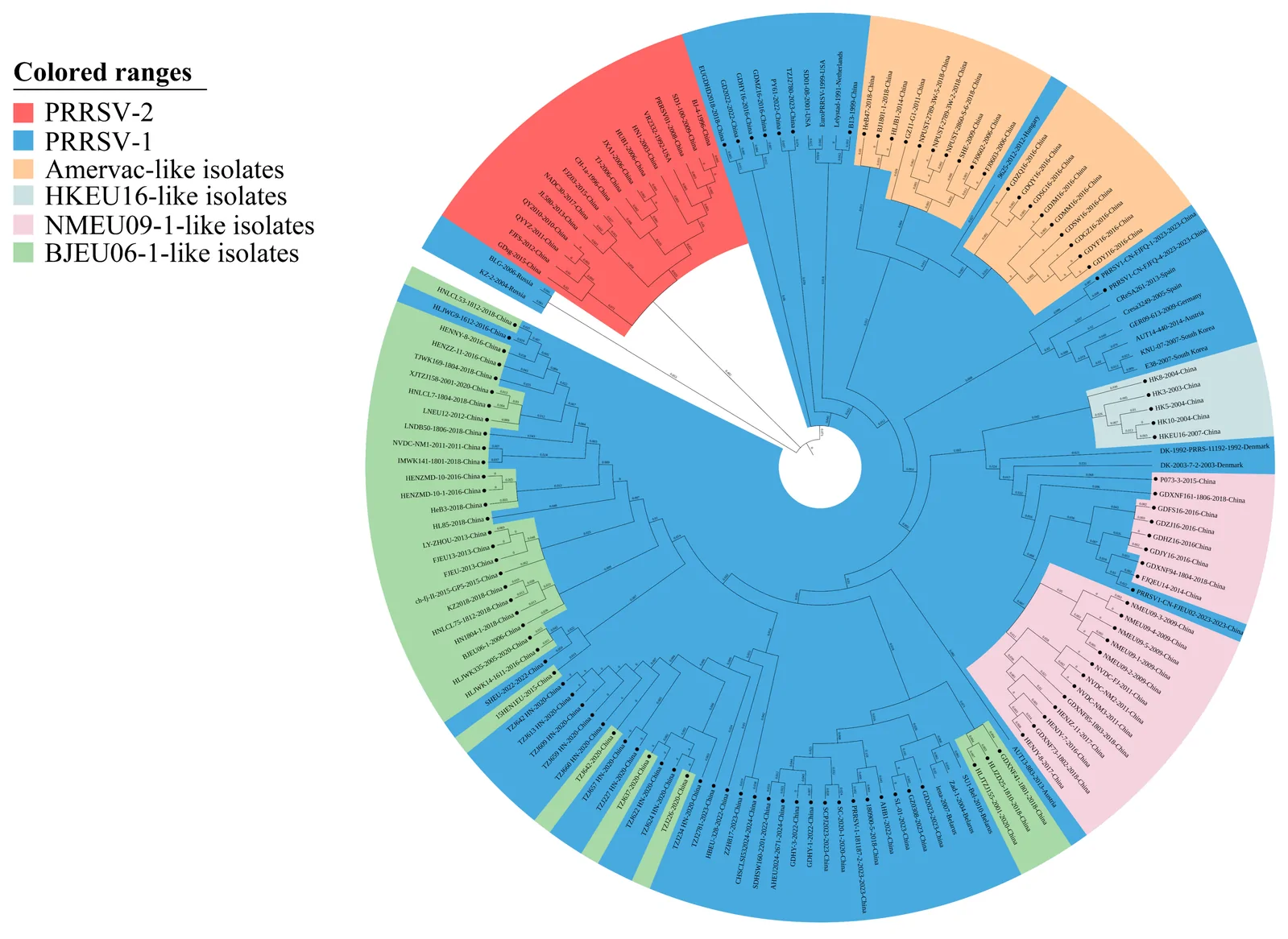
Phylogenetic analysis of PRRSV GP5 gene using the Maximum Likelihood (ML) method. The tree was constructed based on 133 PRRSV-1 and 16 PRRSV-2 isolates. PRRSV-2 and PRRSV-1 lineages are highlighted with red and blue backgrounds, respectively. Chinese PRRSV-1 isolates are marked with circles (●). Numbers at the nodes represent branch lengths.

Phylogenetic tree analysis revealed that the 149 strains used in this study were classified into two distinct clades: PRRSV-1 and PRRSV-2 (Figure 3 and Figure 4). The PRRSV-1 clade was further divided into two subclades, with the larger subclade being categorized into four lineages: Amervac-like [21], BJEU06-1-like, HKEU16-like, and NMEU09-1-like [22]. These four lineages were defined based on ORF5 phylogenetic analysis using both NJ and ML methods with 1000 bootstrap replicates, following the classification framework established in previous studies on Chinese PRRSV-1 [21,22]. Among these, BJEU06-1-like strains constitute the predominant epidemic subgroup in China, with NMEU09-1-like as the secondary subgroup [3,12]. PRRSV-1 has now been detected in at least 28 regions of China, and Chinese isolates have been classified into multiple subgroups within the Western European subtype I [12]. In the maximum likelihood (ML) phylogenetic tree, strains HeB47 and BJ1801-1 from the Amervac-like lineage formed a clade that was topologically closer to PRRSV-2 than to other PRRSV-1 lineages, while strains within the BJEU06-1-like lineage were most closely associated with PRRSV-1. In the neighbor-joining (NJ) phylogenetic tree, strains of the BJEU06-1-like lineage were most closely related to PRRSV-1, whereas strains PY61 and TZJ2780 from the NMEU09-1-like lineage showed closer phylogenetic affinity to PRRSV-2. Furthermore, a relatively close phylogenetic relationship was observed between the BJEU06-1-like and NMEU09-1-like lineages. It is noteworthy that the European representative strains Lelystad virus (LV) andB13 were consistently positioned within the same evolutionary branch in both phylogenetic trees.

### 3.4. Bayesian Evolutionary Dynamics and tMRCA Estimates

A Bayesian evolutionary analysis was conducted on the 114 Chinese PRRSV-1 GP5 sequences. The evolutionary rate was estimated to be 1.94 × 10^−3^ substitutions per site per year (95% highest posterior density [HPD] interval: 1.17 × 10^−3^–2.71 × 10^−3^), with an effective sample size (ESS) of 10,577. The tMRCA of all Chinese PRRSV-1 GP5 sequences was estimated to be approximately 1910 (95% HPD: 1864.31–1952.97), indicating that the sampled strains shared a common ancestor dating to the early 20th century. This estimate reflects the inferred time at which the ancestral sequence of the sampled strains existed, rather than the time of introduction of PRRSV-1 into China. Temporal signals were assessed, supporting the use of a strict molecular clock model for this dataset and confirming that the sequences contain sufficient temporal structure for reliable inference of evolutionary dynamics. However, it should be noted that clock model selection was based on marginal likelihood comparisons, and the strict clock assumption may not hold universally across all lineages.

**Figure 5:**
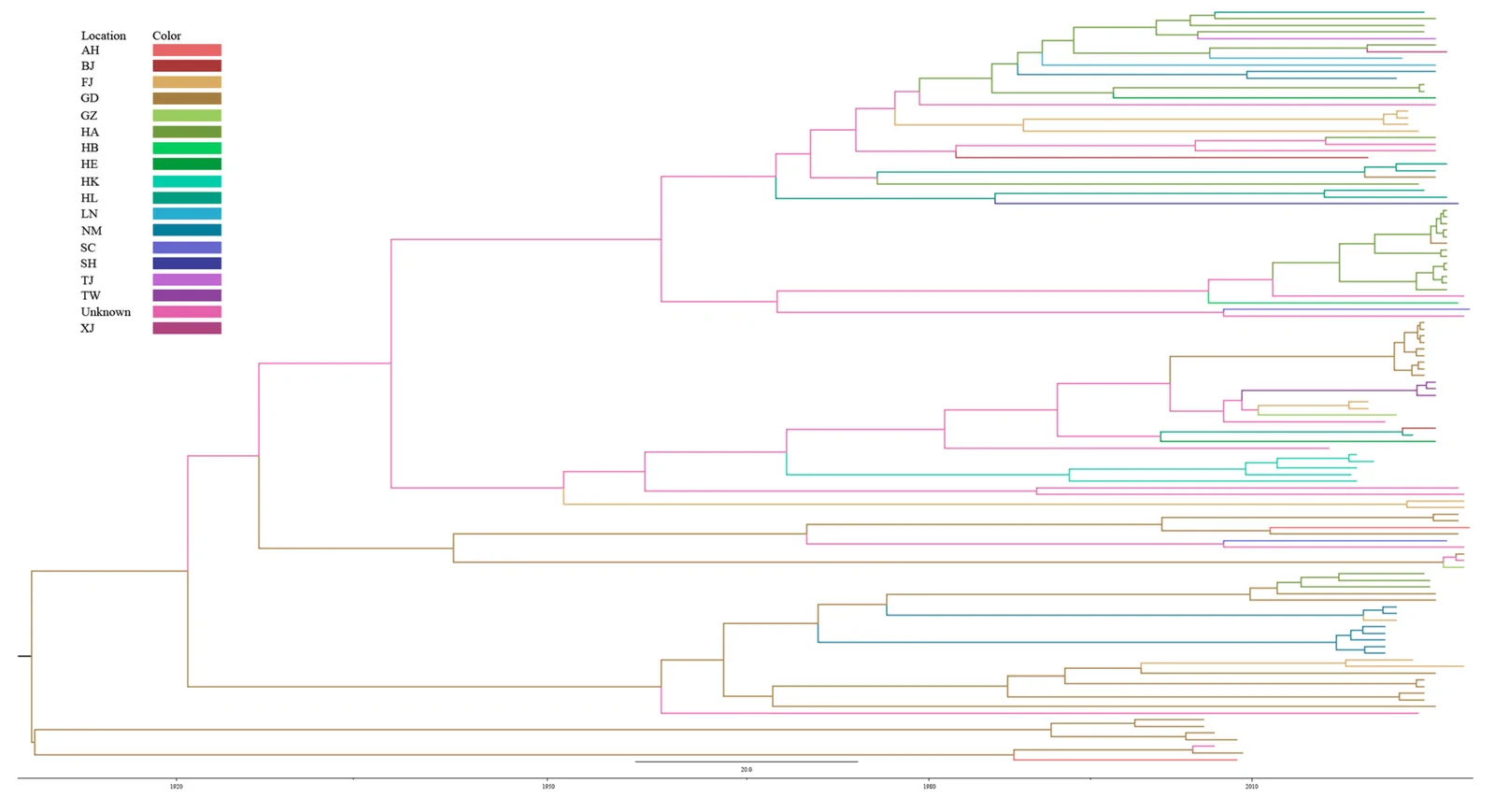
Time-scaled phylogenetic tree of Chinese PRRSV-1 GP5 sequences. Branch colors indicate geographic origin (see color legend inset). The *x*-axis shows time in calendar years, with the tMRCA estimated at ~1910 (95% HPD: 1864–1953). Posterior probabilities (≥0.70) are shown at key nodes.

Based on the time-scaled phylogenetic tree reconstructed using Bayesian inference (Figure 5), the evolutionary history and temporal evolutionary patterns and geographic distribution of Chinese PRRSV-1 GP5 sequences were investigated. The maximum clade credibility (MCC) tree revealed that all 114 Chinese PRRSV-1 strains shared a common ancestor. The tree also revealed that certain lineages (Amervac-like and NMEU09-1-like) exhibited longer branch lengths, indicative of higher substitution rates or longer circulation periods. Overall, the time-scaled phylogenetic tree revealed the temporal evolutionary relationships among Chinese PRRSV-1 GP5 sequences and illustrated the geographic distribution of different lineages, suggesting complex regional circulation patterns. However, we note that formal phylogeographic analyses would be required to rigorously infer regional lineage distribution dynamics.

### 3.5. Recombination Events in the GP5 Gene

Based on multiple sequence alignment analysis of GP5 sequences derived from 114 PRRSV genomes, no significant recombination events were detected within the GP5 gene among the Chinese PRRSV-1 strains analyzed. This observation is consistent with previous reports indicating that ORF5 generally exhibits limited recombination activity in PRRSV [23].

### 3.6. Predicted Structural and Antigenic Features of GP5

#### 3.6.1. Predicted Transmembrane Topology of GP5

**Figure 6:**
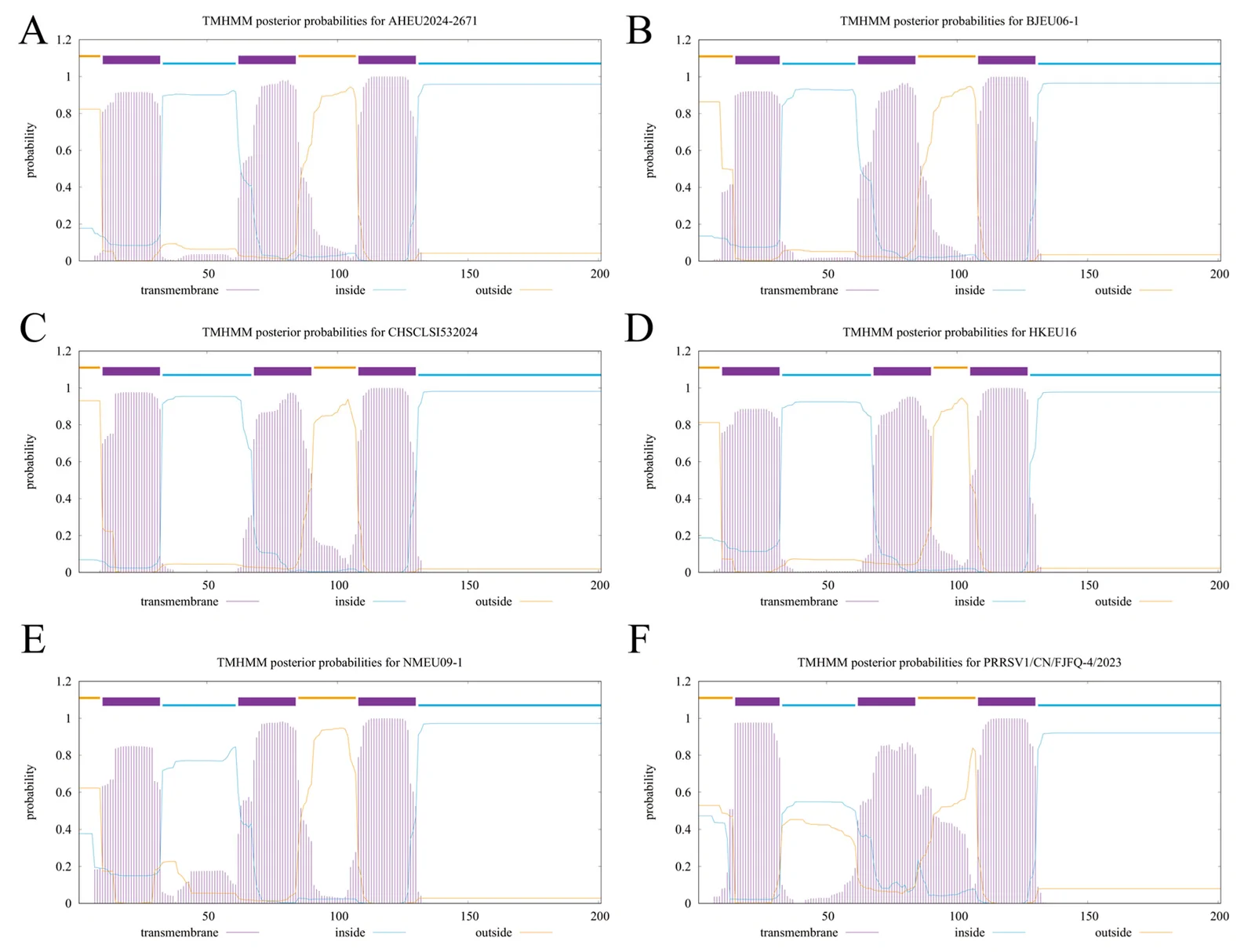
Prediction of Transmembrane Domains in PRRSV-1 GP5. (**A**) AHEU2024-2671, (**B**) BJEU06-1, (**C**) CHSCLSI532024, (**D**) HKEU16, (**E**) NMEU09-1, (**F**) PRRSV/CN/FJFQ-4/2023. The predicted transmembrane helices are shown as red peaks above the threshold line. The *x*-axis represents amino acid position, and the *y*-axis represents the probability of transmembrane localization. Three conserved transmembrane domains (TM1, TM2, TM3) were identified in all six sequences.

The transmembrane structure prediction of the GP5 protein from six Chinese PRRSV-1 strains revealed that these sequences all exhibit highly consistent topological characteristics. Three typical transmembrane domains (TMDs)—TM1, TM2, and TM3—were identified in all sequences, with nearly identical positions, indicating that the transmembrane architecture of GP5 is highly conserved among these strains. The N-terminus was consistently localized extracellularly, while the C-terminus was positioned intracellularly. Clear inside/outside probability transitions were observed in the transmembrane regions, supporting a stable membrane-spanning mode. Although minor variations were noted in the width or steepness of the transmembrane probability curves in certain regions among different strains, these did not affect the number of TMDs or the overall topology, suggesting only local amino acid divergence among sequences. Overall, no significant variation in transmembrane structure was detected in the six Chinese PRRSV-1 GP5 sequences, and the characteristic triple-transmembrane architecture typical of European-type PRRSV-1 GP5 was maintained (Figure 6).

#### 3.6.2. Predicted N-Glycosylation Patterns of GP5

**Figure 7:**
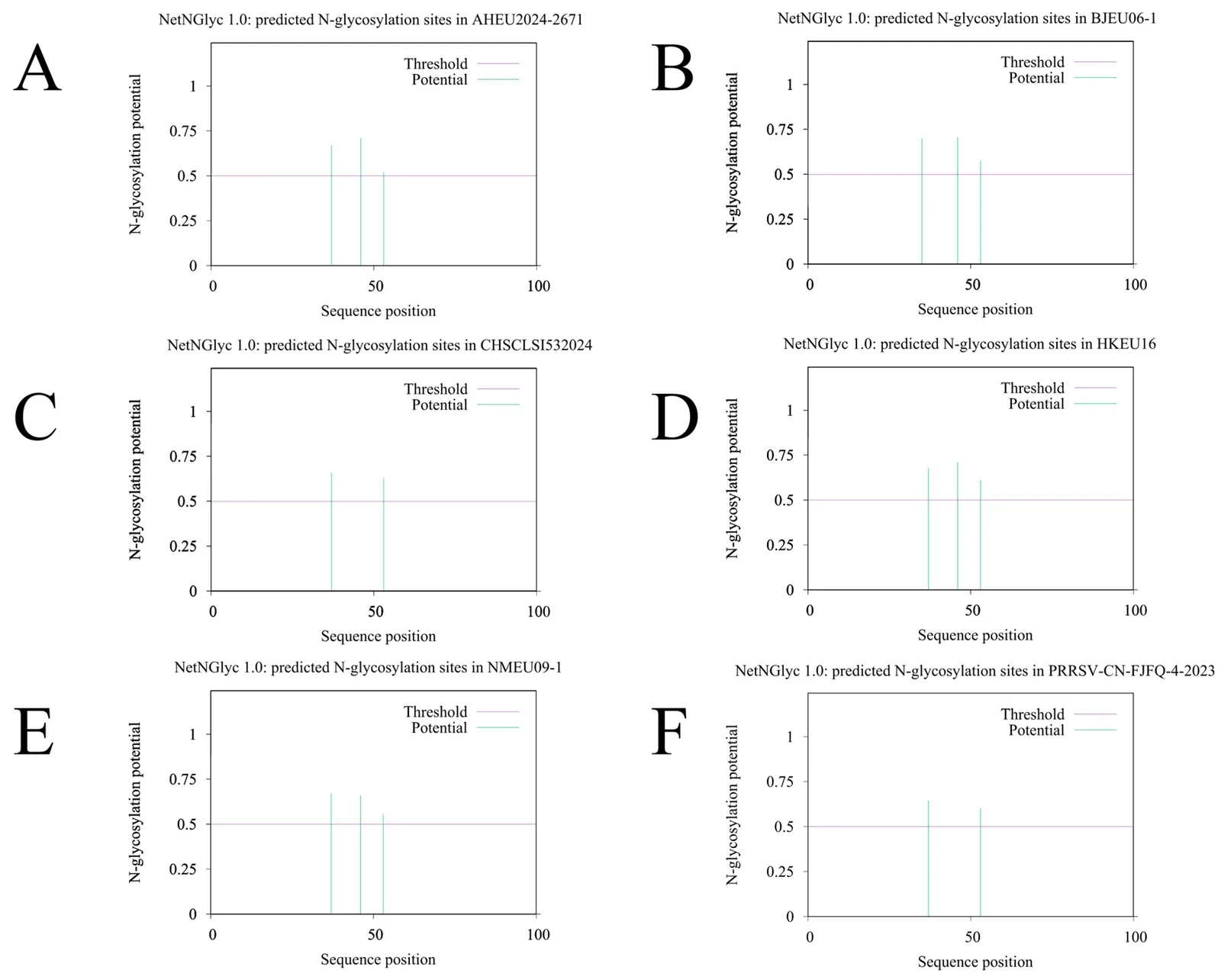
Prediction of N-Glycosylation Sites in PRRSV-1 GP5. (**A**) AHEU2024-2671, (**B**) BJEU06-1, (**C**) CHSCLSI532024, (**D**) HKEU16, (**E**) NMEU09-1, (**F**) PRRSV/CN/FJFQ-4/2023. Predictions were performed using NetNGlyc 1.0 with a default threshold of 0.5; peaks above the threshold indicate potential N-glycosylation sites. The *x*-axis represents amino acid position, and the *y*-axis represents the glycosylation potential score.

Based on the N-glycosylation site prediction results of six Chinese PRRSV-1 GP5 amino acid sequences (Figure 7), similar glycosylation characteristics were observed across all sequences. Potential N-glycosylation sites were predominantly identified in the N-terminal region, approximately between positions 30–60, with all predicted peaks significantly exceeding the threshold, indicating a high likelihood of glycosylation at these sites. Although minor variations in the number and intensity of peaks were noted among different strains, a consistent pattern of 2–3 stable potential glycosylation sites were revealed, suggesting a degree of conservation in the glycosylation pattern of the GP5 protein among PRRSV-1 strains. These predicted sites correspond to the classical N-X-S/T motif previously reported for GP5 in existing studies [24,25], indicating their potential functional importance in viral assembly, immune evasion, and antigenic structural stability. Overall, a consistent distribution of glycosylation sites was maintained among the sequences, reflecting structural conservation in the glycosylation characteristics of GP5 among Chinese PRRSV-1 strains.

#### 3.6.3. AlphaFold3-Predicted Tertiary Structures of GP5

**Figure 8:**
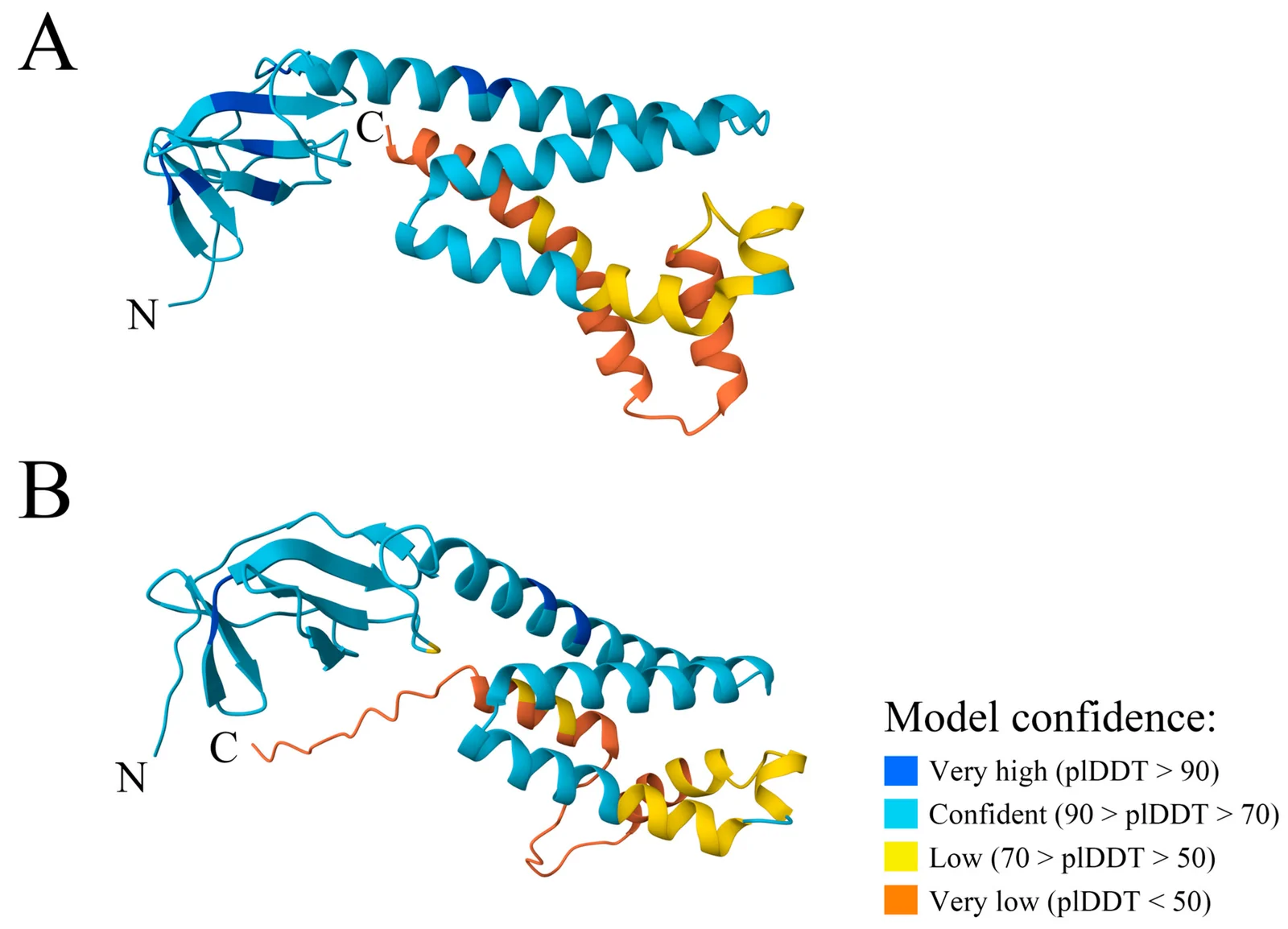
Prediction of the GP5 tertiary structure. (**A**) VR2332, (**B**) BJEU06-1. In the predicted structures, varying levels of model confidence are represented by distinct colors (blue, cyan, yellow, and orange for very high, high, low, and very low confidence, respectively) These confidence levels are determined by the predicted Local Distance Difference Test (pLDDT) score. The pLDDT score, ranging from 0 to 100, is utilized to evaluate the per-residue prediction reliability, with higher values indicating greater structural accuracy. The N- and C-terminus of the proteins are indicated by N and C, respectively.

Based on the structural predictions generated by AlphaFold3 for the GP5 proteins of two PRRSV strains (Figure 8), both were characterized by a typical conformation composed of an N-terminal β-sheet domain and a C-terminal helical bundle, though distinct differences were observed in the local predicted confidence levels and spatial arrangements. Higher model confidence was displayed in the N-terminal regions of both GP5 proteins, suggesting relatively stable folding in these parts. This observation appears to contradict the well-documented sequence hypervariability of the GP5 N-terminus. Such a paradox can be explained by the genomic overlap between the GP5-encoding region and ORF5a, which encodes a conserved arginine/glutamine-rich motif that imposes structural constraints on the overlapping reading frame while permitting amino acid diversity in GP5. Furthermore, the presence of variable N-glycosylation sites in the N-terminal domain allows sequence changes to be accommodated without disrupting the overall fold. Consequently, despite the high sequence diversity, the N-terminal three-dimensional scaffold is maintained, as reflected by the high confidence scores. In contrast, large proportions of the flexible loops and peripheral helices extending from the transmembrane regions were assigned low or very low confidence scores in both strains, consistent with the possibility that these regions are structurally flexible, although such interpretations remain speculative due to the low confidence of the predictions [26]. When the two structures were compared, a more continuous distribution of high-confidence regions was shown in the central helical domain of BJEU06-1, whereas VR2332 was characterized by a longer low-confidence segment in the corresponding region, implying possible sequence-driven flexibility differences between them. Additionally, slight differences were noted in the helical arrangement of their C-terminal regions, although the low confidence scores in this region preclude firm conclusions regarding functional implications. Overall, the AlphaFold3 predictions revealed local conformational variations within the conserved structural framework of PRRSV-1 and PRRSV-2 GP5 (Figure 8), providing a structural basis for further investigation of their functional divergence and antigenic properties.

#### 3.6.4. Predicted B-Cell Epitopes in GP5

The B-cell linear epitope prediction results for the six selected Chinese PRRSV-1 GP5 sequences (Figure 7) revealed a high degree of consistency in overall trends. Typical epitope-rich region characteristics of the PRRSV GP5 protein were observed across all sequences, with prominent antigenicity peaks identified in the extracellular regions, while relatively low scores were generally assigned to the transmembrane domains and their adjacent areas.

In all profiles, high-scoring segments were predominantly concentrated in the N-terminal ectodomain of GP5, suggesting that this region may represent an important immunodominant epitope commonly conserved among multiple PRRSV-1 strains. Simultaneously, minor fluctuations were noted among the sequences, with subtle variations observed in the peak height and range of high-scoring segments in certain strains, reflecting antigenic determinant variability across different isolates.

**Figure 9:**
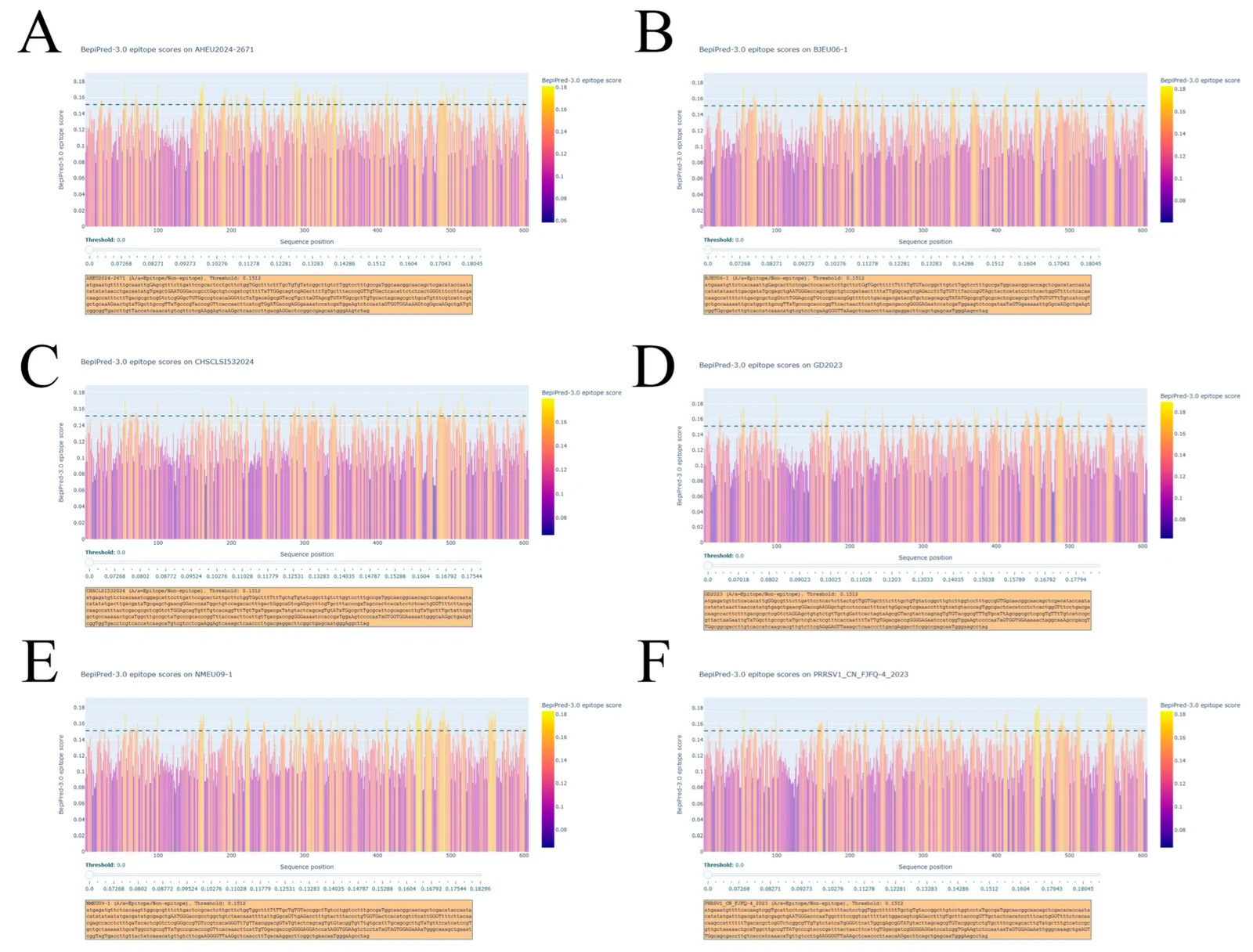
Prediction of B-cell Epitopes in PRRSV-1 GP5. (**A**) AHEU2024-2671, (**B**) BJEU06-1, (**C**) CHSCLSI532024, (**D**) GD2023, (**E**) NMEU09-1, (**F**) PRRSV/CN/FJFQ-4/2023. Predictions were performed using BepiPred-3.0 with a threshold of 0.1512 (dashed line); scores above the threshold indicate predicted epitope residues. The *x*-axis represents amino acid position, and the *y*-axis represents the epitope propensity score. The amino acid sequences at the bottom correspond to the GP5 sequence of each strain.

These differences may potentially affect antibody recognition efficiency, thereby contributing to immune evasion processes to some extent. Overall, the B-cell linear epitope predictions for the six GP5 sequences (Figure 9) indicated that the core antigenic segment structure remains relatively conserved, while strain-specific variations are still present and should be considered in subsequent research and vaccine design.

## 4. Discussion

We systematically characterized the genetic diversity, lineage composition, structural characteristics, and antigenic features of PRRSV-1 GP5 using sequences collected in China between 1999 and 2024. This analysis established a foundational genetic framework for the gene. The overall GP5 architecture was found to preserve the canonical triple-transmembrane structure and the epitope-enriched N-terminal region typical of PRRSV. However, substantial genetic variation was identified, with mutation and deletion hotspots being predominantly concentrated within the N-terminal hypervariable region. By phylogenetic analysis, the Chinese PRRSV-1 strains were further classified into four major lineages: Amervac-like, BJEU06-1-like, HKEU16-like, and NMEU09-1-like, which is consistent with previously reported PRRSV-1 lineages circulating in China [12]. A topological proximity of several recent Chinese isolates (e.g., HeB47, BJ1801-1) to PRRSV-2 strains was observed in the phylogenetic trees, suggesting a possible evolutionary affinity that warrants further investigation. We emphasize that this observation is preliminary and does not constitute evidence for true phylogenetic clustering, which would require formal analyses of homoplasy, site-specific convergent substitutions, and selection pressures using whole-genome data [27,28]. It should be noted, however, that this observation is based primarily on phylogenetic proximity rather than formal tests of convergent substitutions, homoplasy, or structural convergence. Therefore, while suggestive, this finding should be interpreted with caution and warrants further investigation using whole-genome data and functional assays.

We acknowledge that formal selection pressure analyses (e.g., dN/dS, FEL, MEME, or FUBAR) were not performed in this study, as our primary aim was to characterize phylogenetic lineages and antigenic features rather than to quantify evolutionary constraints. Nevertheless, to further explore the observed phylogenetic clustering of certain PRRSV-1 isolates (e.g., HeB47, BJ1801-1) with PRRSV-2, we examined their amino acid sequences at key positions. However, no consistent PRRSV-2-specific amino acid motifs, deletions, or glycosylation patterns were uniquely shared by these clustering isolates, suggesting that the topological proximity observed in the GP5 tree may not reflect functional convergence at the protein level. Future studies employing full-genome sequences and formal selection pressure tests are required to determine whether this phylogenetic signal represents genuine adaptive convergence or other evolutionary phenomena.

The evolutionary dynamics were further clarified through Bayesian analysis. A temporal signal was detected, supporting a clock-like evolutionary pattern in the Chinese PRRSV-1 GP5 dataset. Nevertheless, given the limited sampling over a relatively short time span (1999–2024) and the reliance on a single gene segment, the estimated evolutionary rate (1.94 × 10^−3^ subs/site/year) should be interpreted with caution and may not be directly generalizable to the entire viral genome. From the time-scaled phylogeny. The phylogeny indicated that all Chinese PRRSV-1 strains shared a most recent common ancestor dating to approximately 1910, while certain lineages exhibited extended branch lengths, which were interpreted as indicative of higher evolutionary rates or longer-term circulation.

Beyond these temporal dynamics, the structural and antigenic implications of the observed genetic variation were further explored. At the structural level, a high degree of conservation was observed in the transmembrane architecture of GP5, whereas the N-terminal region exhibited pronounced variability, consistent with the observed sequence heterogeneity and differences in structural confidence. Glycosylation patterns were generally found to be conserved, although strain-specific variations were detected. Such variations are predicted to potentially influence antibody accessibility. However, this remains a working hypothesis that requires direct validation through neutralizing antibody assays using isolate-specific sera or monoclonal antibody competition experiments. Similarly, from the B-cell linear epitope predictions, a conserved core antigenic structure within the N-terminal ectodomain was revealed, accompanied by subtle strain-dependent differences. These antigenic variations, together with potential conformational differences between PRRSV-1 and PRRSV-2 GP5 proteins, are likely to contribute to antigenic drift [29,30]. Therefore, continuous monitoring of GP5 evolution may provide valuable molecular and antigenic information to support vaccine design, although experimental validation of cross-protection remains indispensable.

Several limitations of this study should be acknowledged. The presence of closely related GP5 sequences across different provinces suggests genetic connectivity between regions, which is likely driven by the movement of live pigs rather than long-distance viral transmission. However, given the limitations of single-gene analysis, our data do not allow direct inference of transmission routes or spread dynamics. First, our analyses were confined to the GP5 gene. While this gene is a key target for immune surveillance, it represents only a small fraction of the PRRSV genome. Consequently, the phylogenetic signals observed, including the topological proximity of some Chinese PRRSV-1 isolates to PRRSV-2, are based on a single, highly variable gene segment and do not constitute evidence for genome-wide or functional convergence. Second, the sampling of publicly available sequences may not fully represent the genetic diversity of PRRSV-1 in all regions of China. Third, systematic comparisons of in vivo and in vitro biological characteristics among the four lineages (Amervac-like, BJEU06-1-like, HKEU16-like, and NMEU09-1-like) are currently lacking. To our knowledge, only the BJEU06-1-like strain ZD-1 has been experimentally evaluated for pathogenicity, showing moderate virulence in piglets [31]. Comprehensive characterization of pathogenic, antigenic, and transmission differences among all four lineages represents an important direction for future research. Future studies employing full-genome sequencing, coupled with formal tests for convergent evolution (e.g., homoplasy-based methods and selection pressure analyses), are necessary to validate and extend our findings. Although no recombination events were detected within this gene, it is well established that recombination hotspots in PRRSV are more frequently located in other genomic regions, such as Nsp2 [32,33]. Consequently, the recombination events frequently reported in other genomic regions fall entirely outside the scope of this study, and no inferences regarding intergenotypic or whole-genome recombination can be drawn from the current dataset. The potential risk of recombination between PRRSV-1 and PRRSV-2, therefore, remains an open question that requires future validation through nationwide whole-genome surveillance. Such integrated approaches are essential to provide a more comprehensive understanding of viral evolution and transmission.

First, and most importantly, all evolutionary and phylogenetic analyses in this study are based solely on the GP5 gene (ORF5). While GP5 is a major envelope protein that carries key neutralizing epitopes and is widely used for PRRSV genotyping, it represents less than 5% of the viral genome. Consequently, our findings—including lineage classification, phylogenetic relationships, and evolutionary rate estimates—are locus-specific and cannot be extrapolated to the entire viral genome. Whole-genome sequencing and analysis are required to comprehensively understand PRRSV-1 evolution, recombination, and population dynamics in China. Additionally, because recombination hotspots in PRRSV are predominantly located in other genomic regions (e.g., Nsp2), the absence of recombination events detected in GP5 does not rule out recombination elsewhere in the genome.

In conclusion, the ongoing evolution and regional dissemination of PRRSV-1 in China, alongside its complex relationship with PRRSV-2, were recognized as presenting persistent challenges. Important molecular insights into the evolution of PRRSV-1 in China were provided by these findings, which may contribute to improved vaccine design and more effective control strategies for PRRS in swine production. Specifically, the identification of lineage-specific mutation patterns in the N-terminal ectodomain and the predicted variations in glycosylation and B-cell epitopes offer testable targets for future vaccine antigen design. To advance control efforts, the establishment of an integrated surveillance network is recommended. Furthermore, interdisciplinary approaches combining immunomics, structural biology, and computational design should be employed to elucidate immune escape mechanisms, as the current silico predictions cannot substitute for experimental evidence. Ultimately, a transition from reactive measures to predictive, precision-based management was deemed crucial for mitigating the economic impact of PRRS.

## 5. Conclusions

We systematically characterized based on sequences collected from 1999 to 2024. Four major lineages were identified among the Chinese strains. A preliminary phylogenetic observation suggested that several recent isolates appeared topologically close to PRRSV-2; however, this may reflect phylogenetic artifacts (e.g., long-branch attraction, homoplasy, or model bias) rather than genuine evolutionary relatedness and requires whole-genome validation. A clock-like pattern was preliminarily indicated, warranting further validation. Bayesian analysis estimated the evolutionary rate at 1.94 × 10^−3^ substitutions per site per year, with a time to the most recent common ancestor dated to approximately 1910. The highly conserved transmembrane topology of GP5 was consistently observed, whereas mutations and deletions were found to be repeatedly concentrated in the N-terminal hypervariable region. These characteristics highlight the necessity for continuous antigenic surveillance of the N-terminal domain [34,35]. A molecular basis is therefore provided for the development of broadly protective vaccines and region-adapted control strategies against PRRSV-1 in China.

## Figures and Tables

**Table 1:** Information on the reference sequence of PRRSV GP5.

Strain	Year	Area	GenBank Accession Number	Type
B13	1999	China	AY633973.1	PRRSV-1
HK3	2003	China	KF287129.1	PRRSV-1
HK10	2004	China	KF287131.1	PRRSV-1
HK5	2004	China	KF287130.1	PRRSV-1
HK8	2004	China	KF287128.1	PRRSV-1
FJ0603	2006	China	HM114313.1	PRRSV-1
FJ0602	2006	China	HM755885.1	PRRSV-1
BJEU06-1	2006	China	GU047344.1	PRRSV-1
HKEU16	2007	China	EU076704.1	PRRSV-1
SHE	2009	China	GQ461593.1	PRRSV-1
NMEU09-5	2009	China	GU047343.1	PRRSV-1
NMEU09-1	2009	China	GU047345.1	PRRSV-1
NMEU09-2	2009	China	GU047340.1	PRRSV-1
NMEU09-3	2009	China	GU047341.1	PRRSV-1
NMEU09-4	2009	China	GU047342.1	PRRSV-1
GZ11-G1	2011	China	KF001144.1	PRRSV-1
NVDC-NM2	2011	China	KC492504.1	PRRSV-1
NVDC-NM3	2011	China	KC492505.1	PRRSV-1
NVDC-FJ	2011	China	KC492506.1	PRRSV-1
NVDC-NM1-2011	2011	China	JX187609.1	PRRSV-1
LNEU12	2012	China	KM196101.1	PRRSV-1
FJEU13	2013	China	KP860912.1	PRRSV-1
FJEU	2013	China	KR048690.1	PRRSV-1
LY-ZHOU	2013	China	KY924476.1	PRRSV-1
HLJB1	2014	China	KT224385.1	PRRSV-1
FJQEU14	2014	China	KP860913.1	PRRSV-1
P073-3	2015	China	MK214314.1	PRRSV-1
ch-fj-II-2015-GP5	2015	China	KT208357.1	PRRSV-1
15HEN1 EU	2015	China	KX967492.1	PRRSV-1
HLJWG9-1612	2016	China	OP302771.1	PRRSV-1
HLJWK14-1611	2016	China	OP302768.1	PRRSV-1
GDQY16	2016	China	MF153494.1	PRRSV-1
GDZQ16	2016	China	MF153500.1	PRRSV-1
GDSG16	2016	China	MF153495.1	PRRSV-1
GDMM16	2016	China	MF153492.1	PRRSV-1
GDJM16	2016	China	MF153490.1	PRRSV-1
GDGZ16	2016	China	MF153487.1	PRRSV-1
GDYJ16	2016	China	MF153498.1	PRRSV-1
GDYF16	2016	China	MF153497.1	PRRSV-1
GDSW16	2016	China	MF153496.1	PRRSV-1
GDHZ16	2016	China	MF153489.1	PRRSV-1
GDJY16	2016	China	MF153491.1	PRRSV-1
GDFS16	2016	China	MF153486.1	PRRSV-1
GDZJ16	2016	China	MF153499.1	PRRSV-1
HENJY-7	2016	China	MG870199.1	PRRSV-1
GDHY16	2016	China	MF153488.1	PRRSV-1
GDMZ16	2016	China	MF153493.1	PRRSV-1
HENNY-8	2016	China	MG870202.1	PRRSV-1
HENZMD-10	2016	China	KY363382.1	PRRSV-1
HENZMD-10-1	2016	China	MG870203.1	PRRSV-1
HENZZ-11	2016	China	MG870204.1	PRRSV-1
HENJZ-11	2017	China	MG870201.1	PRRSV-1
HENJY-8	2017	China	MG870200.1	PRRSV-1
IMWK141-1801	2018	China	OP302776.1	PRRSV-1
HNLCL7-1804	2018	China	OP302774.1	PRRSV-1
TJWK169-1804	2018	China	OP302773.1	PRRSV-1
HNLCL53-1812	2018	China	OP302772.1	PRRSV-1
LNDB50-1806	2018	China	OP302770.1	PRRSV-1
HNLCL75-1812	2018	China	OP302767.1	PRRSV-1
GDXNF41-1801	2018	China	OP302766.1	PRRSV-1
HLJZD25-1810	2018	China	OP302764.1	PRRSV-1
GDXNF73-1802	2018	China	OP302762.1	PRRSV-1
GDXNF85-1803	2018	China	OP302761.1	PRRSV-1
GDXNF161-1806	2018	China	OP302759.1	PRRSV-1
GDXNF94-1804	2018	China	OP302758.1	PRRSV-1
EUGDHD2018	2018	China	MK639926.1	PRRSV-1
NPUST-2789-3W-2	2018	China	MN242825.1	PRRSV-1
NPUST-2789-3W-5	2018	China	MN265857.1	PRRSV-1
NPUST-2860-S-6	2018	China	MN265858.1	PRRSV-1
BJ1801-1	2018	China	MK689109.1	PRRSV-1
HeB47	2018	China	MN927228.1	PRRSV-1
HeB3	2018	China	MN927227.1	PRRSV-1
HL85	2018	China	MN927229.1	PRRSV-1
KZ2018	2018	China	MN550991.1	PRRSV-1
HN1804-1	2018	China	MK689121.1	PRRSV-1
180900-5	2018	China	MK303390.1	PRRSV-1
XJTZJ158-2001	2020	China	OP302775.1	PRRSV-1
HLJWK335-2005	2020	China	OP302769.1	PRRSV-1
HLJTZJ155-2001	2020	China	PP330950.1	PRRSV-1
TZJ642	2020	China	OP302763.1	PRRSV-1
TZJ624_HN	2020	China	OQ920890.1	PRRSV-1
TZJ622_HN	2020	China	OQ920889.1	PRRSV-1
TZJ234_HN	2020	China	OQ920888.1	PRRSV-1
TZJ227_HN	2020	China	OQ920887.1	PRRSV-1
TZJ657_HN	2020	China	OQ920886.1	PRRSV-1
TZJ642_HN	2020	China	OQ920885.1	PRRSV-1
TZJ609_HN	2020	China	OQ920884.1	PRRSV-1
TZJ613_HN	2020	China	OQ920883.1	PRRSV-1
TZJ659_HN	2020	China	OQ920882.1	PRRSV-1
TZJ660_HN	2020	China	OQ920881.1	PRRSV-1
TZJ637	2020	China	OP566683.1	PRRSV-1
TZJ226	2020	China	OP566682.1	PRRSV-1
SC-2020-1	2020	China	MW115431.1	PRRSV-1
SDHSW160-2201	2022	China	OP302760.1	PRRSV-1
SHEU-2022	2022	China	PQ306310.1	PRRSV-1
GDHY-1	2022	China	PP315356.1	PRRSV-1
GDHY-3	2022	China	PP315355.1	PRRSV-1
AHB1	2022	China	PP068352.1	PRRSV-1
HBEU-328	2022	China	OR636058.1	PRRSV-1
PY61	2022	China	PP402111.1	PRRSV-1
GD2022	2022	China	OQ606399.1	PRRSV-1
PRRSV1/CN/FJFQ-4/2023	2023	China	OR502390.1	PRRSV-1
SCPJ2023	2023	China	PP800763.1	PRRSV-1
SL-01	2023	China	OQ871594.1	PRRSV-1
GD2023	2023	China	PP556438.1	PRRSV-1
TZJ2780	2023	China	PP341289.1	PRRSV-1
TZJ2781	2023	China	PP341290.1	PRRSV-1
ZZH817	2023	China	PP350850.1	PRRSV-1
PRRSV1/CN/FJFQ-1/2023	2023	China	OR260421.1	PRRSV-1
PRRSV1/CN/FJEU02/2023	2023	China	PQ553567.1	PRRSV-1
PRRSV-1/181187-2/2023	2023	China	OQ856755.1	PRRSV-1
GZ0308	2023	China	PQ338766.1	PRRSV-1
AHEU2024-2671	2024	China	PQ640355.1	PRRSV-1
CHSCLSI532024	2024	China	PQ857580.1	PRRSV-1
Lelystad virus	1991	Netherlands	M96262.2	PRRSV-1
DK-1992-PRRS-111_92	1992	Denmark	KC862566.1	PRRSV-1
EuroPRRSV	1999	USA	AY366525.1	PRRSV-1
SD01-08	2001	USA	DQ489311.1	PRRSV-1
DK-2003-7-2	2003	Denmark	KC862572.1	PRRSV-1
KZ-2	2004	Russia	EU071239.1	PRRSV-1
Zad-1	2004	Belarus	DQ324694.2	PRRSV-1
Cresa3249	2005	Spain	JF276433.1	PRRSV-1
BLG	2006	Russia	EU071232.1	PRRSV-1
Cresa3267	2006	Portugal	JF276435.1	PRRSV-1
KNU-07	2007	South Korea	FJ349261.1	PRRSV-1
E38	2007	South Korea	KT033457.1	PRRSV-1
lena	2007	Belarus	JF802085.1	PRRSV-1
GER09-613	2009	Germany	KT344816.1	PRRSV-1
SU1-Bel	2010	Belarus	KP889243.1	PRRSV-1
9625/2012	2012	Hungary	KJ415276.1	PRRSV-1
CReSA261	2013	Spain	KX249756.1	PRRSV-1
AUT13-883	2013	Austria	KT326148.1	PRRSV-1
AUT14-440	2014	Austria	KT334375.1	PRRSV-1
VR2332	1992	USA	EF536003.1	PRRSV-2
BJ-4	1996	China	AF331831.1	PRRSV-2
CH-1a	1996	China	AY032626.1	PRRSV-2
HN1	2003	China	AY457635.1	PRRSV-2
JXA1	2006	China	EF112445.1	PRRSV-2
TJ	2006	China	EU860248.1	PRRSV-2
HUB1	2006	China	EF075945.1	PRRSV-2
PRRSV01	2008	China	FJ175687.1	PRRSV-2
SD1-100	2009	China	GQ914997.1	PRRSV-2
QY2010	2010	China	JQ743666.1	PRRSV-2
QYYZ	2011	China	JQ308798.1	PRRSV-2
FJFS	2012	China	KP998476.1	PRRSV-2
JL580	2013	China	KR706343.1	PRRSV-2
GDsg	2015	China	KX621003.1	PRRSV-2
FJZ03	2015	China	KP860909.1	PRRSV-2
NADC30	2017	China	MH500776.1	PRRSV-2

**Table 2:** Nucleotide and amino acid sequence similarities among the PRRSV-1 GP5 sequences analysed in this study (114 Chinese + 19 non-Chinese isolates).

Sequence Type	Overall Range	Highest Similarity (Strain Pair)	Lowest Similarity (Strain Pair)
Nucleotide	60.03–100%	100% (FJEU vs. FJEU13)	60.03% (GDXNF85-1803 vs. SU1-Bel)
Amino acid	75.90–100%	100% (multiple pairs; e.g., FJEU13 vs. FJEU, FJ0602 vs. FJ0603)	75.90% (HENJZ-11-2017 vs. Zad-1)

Note: The highest and lowest values were derived from pairwise comparisons of all 133 PRRSV-1 isolates. For nucleotide sequences, the lowest similarity between Chinese and non-Chinese strains was 60.03% (GDXNF85-1803 vs. SU1-Bel); within the Chinese isolates alone, the lowest nucleotide similarity was 79.42% (GDXNF85-1803 vs. B13). For amino acid sequences, the lowest similarity overall (75.90%) occurred between the Chinese strain HENJZ-11-2017 and the Belarusian strain Zad-1; among Chinese strains only, the lowest amino acid similarity was 79.49% (TZJ2780 vs. ZZH817). The highest amino acid similarity between Chinese and non-Chinese isolates was 98.97% (B13 vs. Lelystad virus).

## Data Availability

All datasets generated for this study are included in the article/Supplementary Materials. The data that support the findings of this study are openly available in Figshare at https://doi.org/10.6084/m9.figshare.30959372.v8 [36].

## Supplementary Materials

The following supporting information can be downloaded at https://www.mdpi.com/article/10.3390/microorganisms14091963/s1: Table S1: Province-level geographic distribution of the 114 Chinese PRRSV-1 isolates analyzed in this study.

## Abbreviations

BIC	Bayesian Information Criterion
ESS	Effective sample size
GP5	Glycoprotein 5
GTR	General Time Reversible (substitution model)
HPD	Highest posterior density
HP-PRRS	Highly pathogenic porcine reproductive and respiratory syndrome
MCC	Maximum clade credibility
MCMC	Markov chain Monte Carlo
ML	Maximum likelihood
NJ	Neighbor-Joining
NSP2	Nonstructural protein 2
ORF	Open reading frame
pLDDT	predicted Local Distance Difference Test
PRRS	Porcine reproductive and respiratory syndrome
PRRSV	Porcine reproductive and respiratory syndrome virus
TMD	Transmembrane domain
TMHMM	Transmembrane Hidden Markov Model (prediction server)
tMRCA	Time to the most recent common ancestor
