# Supplementary material for: Genetic Diversity, Phylogenetic Lineages, and Antigenic Features of the PRRSV-1 GP5 Gene in China
Source: Microorganisms. 2026 Sep 5;14(9):1963. doi: 10.3390/microorganisms14091963 (PMC13608930; doi:10.3390/microorganisms14091963)
Supplement: Supplementary file 1 [file microorganisms-14-01963-s001.zip › microorganisms-4470734-supplementary.pdf]

Table S1. Province-level geographic distribution of the 114 Chinese PRRSV-1 isolates analyzed in this study.

Strain	Year	Province	GenBank Accession Number
B13	1999	Guangdong	AY633973.1
HK3	2003	Hong Kong	KF287129.1
HK10	2004	Hong Kong	KF287131.1
HK5	2004	Hong Kong	KF287130.1
HK8	2004	Hong Kong	KF287128.1
FJ0603	2006	Fujian	HM114313.1
FJ0602	2006	Fujian	HM755885.1
BJEU06-1	2006	Beijing	GU047344.1
HKEU16	2007	Hong Kong	EU076704.1
SHE	2009	Shanghai	GQ461593.1
NMEU09-5	2009	Nei Mongol	GU047343.1
NMEU09-1	2009	Nei Mongol	GU047345.1
NMEU09-2	2009	Nei Mongol	GU047340.1
NMEU09-3	2009	Nei Mongol	GU047341.1
NMEU09-4	2009	Nei Mongol	GU047342.1
GZ11-G1	2011	Guizhou	KF001144.1
NVDC-NM2	2011	Nei Mongol	KC492504.1
NVDC-NM3	2011	Nei Mongol	KC492505.1
NVDC-FJ	2011	Fujian	KC492506.1
NVDC-NM1-2011	2011	Nei Mongol	JX187609.1
LNEU12	2012	Liaoning	KM196101.1
FJEU13	2013	Fujian	KP860912.1
FJEU	2013	Fujian	KR048690.1
LY-ZHOU	2013	Fujian	KY924476.1
HLJB1	2014	Heilongjiang	KT224385.1
FJQEU14	2014	Fujian	KP860913.1
P073-3	2015	Unknown	MK214314.1
ch-fj-II-2015-GP5	2015	Fujian	KT208357.1
15HEN1 EU	2015	Henan	KX967492.1
HLJWG9-1612	2016	Heilongjiang	OP302771.1
HLJWK14-1611	2016	Heilongjiang	OP302768.1
GDQY16	2016	Guangdong	MF153494.1
GDZQ16	2016	Guangdong	MF153500.1
GDSG16	2016	Guangdong	MF153495.1
GDMM16	2016	Guangdong	MF153492.1
GDJM16	2016	Guangdong	MF153490.1
GDGZ16	2016	Guangdong	MF153487.1
GDYJ16	2016	Guangdong	MF153498.1
GDYF16	2016	Guangdong	MF153497.1
GDSW16	2016	Guangdong	MF153496.1

Strain	Year	Province	GenBank Accession Number
GDHZ16	2016	Guangdong	MF153489.1
GDJY16	2016	Guangdong	MF153491.1
GDFS16	2016	Guangdong	MF153486.1
GDZJ16	2016	Guangdong	MF153499.1
HENJY-7	2016	Henan	MG870199.1
GDHY16	2016	Guangdong	MF153488.1
GDMZ16	2016	Guangdong	MF153493.1
HENNY-8	2016	Henan	MG870202.1
HENZMD-10	2016	Henan	KY363382.1
HENZMD-10-1	2016	Henan	MG870203.1
HENZZ-11	2016	Henan	MG870204.1
HENJZ-11	2017	Henan	MG870201.1
HENJY-8	2017	Henan	MG870200.1
IMWK141-1801	2018	Nei Mongol	OP302776.1
HNLCL7-1804	2018	Henan	OP302774.1
TJWK169-1804	2018	Tianjin	OP302773.1
HNLCL53-1812	2018	Henan	OP302772.1
LNDB50-1806	2018	Liaoning	OP302770.1
HNLCL75-1812	2018	Henan	OP302767.1
GDXNF41-1801	2018	Guangdong	OP302766.1
HLJZD25-1810	2018	Heilongjiang	OP302764.1
GDXNF73-1802	2018	Guangdong	OP302762.1
GDXNF85-1803	2018	Guangdong	OP302761.1
GDXNF161-1806	2018	Guangdong	OP302759.1
GDXNF94-1804	2018	Guangdong	OP302758.1
EUGDHD2018	2018	Guangdong	MK639926.1
NPUST-2789-3W-2	2018	Taiwan	MN242825.1
NPUST-2789-3W-5	2018	Taiwan	MN265857.1
NPUST-2860-S-6	2018	Taiwan	MN265858.1
BJ1801-1	2018	Beijing	MK689109.1
HeB47	2018	Hebei	MN927228.1
HeB3	2018	Hebei	MN927227.1
HL85	2018	Unknown	MN927229.1
KZ2018	2018	Unknown	MN550991.1
HN1804-1	2018	Unknown	MK689121.1
180900-5	2018	Unknown	MK303390.1
XJTZJ158-2001	2020	Xinjiang	OP302775.1
HLJWK335-2005	2020	Heilongjiang	OP302769.1
HLJTZJ155-2001	2020	Heilongjiang	PP330950.1
TZJ642	2020	Guangdong	OP302763.1
TZJ624_HN	2020	Henan	OQ920890.1
TZJ622_HN	2020	Henan	OQ920889.1

Strain	Year	Province	GenBank Accession Number
TZJ234_HN	2020	Henan	OQ920888.1
TZJ227_HN	2020	Henan	OQ920887.1
TZJ657_HN	2020	Henan	OQ920886.1
TZJ642_HN	2020	Henan	OQ920885.1
TZJ609_HN	2020	Henan	OQ920884.1
TZJ613_HN	2020	Henan	OQ920883.1
TZJ659_HN	2020	Henan	OQ920882.1
TZJ660_HN	2020	Henan	OQ920881.1
TZJ637	2020	Henan	OP566683.1
TZJ226	2020	Henan	OP566682.1
SC-2020-1	2020	Sichuan	MW115431.1
SDHSW160-2201	2022	Shandong	OP302760.1
SHEU-2022	2022	Shanghai	PQ306310.1
GDHY-1	2022	Guangdong	PP315356.1
GDHY-3	2022	Guangdong	PP315355.1
AHB1	2022	Anhui	PP068352.1
HBEU-328	2022	Hubei	OR636058.1
PY61	2022	Unknown	PP402111.1
GD2022	2022	Guangdong	OQ606399.1
PRRSV1/CN/FJFQ-4/2023	2023	Fujian	OR502390.1
SCPJ2023	2023	Sichuan	PP800763.1
SL-01	2023	Unknown	OQ871594.1
GD2023	2023	Guangdong	PP556438.1
TZJ2780	2023	Unknown	PP341289.1
TZJ2781	2023	Unknown	PP341290.1
ZZH817	2023	Unknown	PP350850.1
PRRSV1/CN/FJFQ-1/2023	2023	Fujian	OR260421.1
PRRSV1/CN/FJEU02/2023	2023	Fujian	PQ553567.1
PRRSV-1/181187-2/2023	2023	Guangdong	OQ856755.1
GZ0308	2023	Guizhou	PQ338766.1
AHEU2024-2671	2024	Anhui	PQ640355.1
CHSCLSI532024	2024	Sichuan	PQ857580.1
